# Synthesis of silane- and quaternary ammonium-bearing copolymers and application as a coating resin on cotton fabric

**DOI:** 10.55730/1300-0527.3616

**Published:** 2023-05-22

**Authors:** Ayşe MARTİN, Nazgül GÜLER, İlknur YERLİ, Hande HANÇER, Tülin ÖZBEK, Tarık EREN

**Affiliations:** 1 Department of Chemistry, Faculty of Arts and Science, Yıldız Technical University, İstanbul, Turkiye; 2 Department of Molecular Biology and Genetics, Faculty of Arts and Science, Yıldız Technical University, İstanbul, Turkiye

**Keywords:** Cationic copolymer, polysiloxanes, fastness properties, dyeing properties, antibacterial activity

## Abstract

In this study, silane and quaternary ammonium functional methacrylate monomers were synthesized and used to construct a copolymer using an emulsion polymerization technique to control the reaction rate. The copolymer was then designed using different ratios of silane and quaternary ammonium groups to investigate the relationship between the structure and properties. The presence of the ethoxy silane group in the copolymer series provided covalent bonding through the silanol group onto cotton fabric. The presence of cationic groups also helped to cover the fabric surface. After coating the cotton textile fabric, the resistance of the dye on the fabric surface to friction was assessed and tests were conducted on washing, rubbing, water, and light fastness. Finally, the textile surfaces were investigated for their antibacterial activity against *Staphylococcus aureus* and *Escherichia coli*. It was observed that the copolymer series showed >99% killing efficiency against *S. aureus* but had no effect on *E. coli*.

## 1. Introduction

The textile industry has changed in recent years with more awareness of and consumer demand for better quality of textile fabrics and garments with higher performance. To survive in this competitive market, garments must provide the quality standards demanded by the operator, especially in terms of the fastness characteristics of the dyed fabric, which is the ability of the fabric to maintain its color [1].

In the textile industry, the value of fabric fastness, which encompasses the effects of washing, water, chlorinated water, light, and rubbing, has become particularly important. In cellulosic fabrics colored with reactive dye, unfixed dye can be washed properly to improve washing fastness; however, it is well known that corresponding improvement in wet-rubbing fastness cannot be easily achieved [2]. When microscopically examined, dyed fabrics are observed to be damaged under wet friction conditions, and microscopically small dye particles may stain the adjacent fabric; therefore, it is very difficult to obtain good wet-rubbing fastness values. In dyed and printed textile materials, unfixed dye particles are held mechanically on the fabric surface and can easily cause staining on the wearer’s skin or any other cloth that comes into contact with the material [2]; therefore, the rubbing fastness of dyed or printed textile materials should be determined. Friction fastness is the transfer by friction of colors from the textile material to other surfaces, and rubbing fastness involves two processes: dry friction and wet friction. In dry friction, the periphery of the colored sample is affected, exposing loose or unfixed paint particles adhering to the surface fibers of the test fabric. The dyestuffs are denser; therefore, there is more staining. In wet friction, uncured dyes are dissolved in water (hydrolysis) and then transferred to the test fabric, resulting in poor wet-rub fastness values. In wet cases, both the color and short colored fibers are transferred to the wearer. As reactive dyes chemically bond with cellulose fibers, their rubbing fastness is expected to be high; however, in reality, the situation is the opposite because the reactive dye in the dye bath becomes hydrolyzed in an alkaline environment. Rubbing fastness can be improved using an effective washing and fixing process [3]; however, wet-rubbing fastness cannot be improved by washing–fixing processes for some colors, such as black or turquoise. In such cases, various chemicals must be used to bring the rubbing fastness to the desired level.

There are several methods discussed in the literature to improve the fastness of fabric through polymer coatings [4]. Kongliang et al. [5] showed that by synthesizing silicone acrylic copolymers containing cationic groups, washing and rubbing fastness improved in fabrics to which the copolymers were applied. In the present study, the ring opening polymerization of the octamethylcyclotetrasiloxane monomer and the radical polymerization method for binding the cationic monomer to octamethylcyclotetrasiloxane were used. The resulting polymer was applied directly to dyed fabric, and it was observed that the silicone acrylate copolymer improved the washing and rubbing fastness beyond that of the fabric with no polymer applied. Kongliang et al. [6] performed a similar study based on a silicone acrylic copolymer bearing cationic functionality. The fabric surface was covered by cationic groups, which increased the resistance of the paint on the fabric surface to friction. It was also observed that introducing silicone functionalities improved the wash fastness and wet-rubbing fastness of the dyed cotton fabric.

In a previous study [7], the synthesis and characterization of a silicone copolymer with hydrophilic properties were described. First, various copolymers were obtained by free radical polymerization using a 3-(tris[trimethylsiloxy]silyl) (TRIS) propyl methacrylate silicon-based monomer. The coated surface was then investigated using a contact angle meter. It was observed that wetting of the silicone surface was not greatly enhanced; however, rubbing fastness was enhanced by introducing the silicone copolymer on the surface of textiles [7].

Miah et al. [8] prepared a modified environmentally friendly silicone polyacrylate binder by emulsion polymerization using soft and hard type functional monomers. The newly synthesized binder was applied to fabric using pigment printing techniques. From the application of this binder, a moderate hand-feel (*T*_g_ = 16.48 °C) was obtained with the fabric having good rubbing fastness properties. The suitable conditions of the reaction, maintenance time, temperature, and chemical dosage helped create a product with excellent properties [8].

Cotton fabrics have long been used in the textile industry. Compared to synthetic fibers, cotton has a large market share in the textile industry thanks to its ease of use and the comfort it provides; however, cotton’s biggest disadvantage is that it provides suitable conditions, such as humidity and temperature, for the growth of bacteria [9,10]. As bacteria reproduces on cotton textile materials, they cause defects, such as odor, color change, and weakening in the mechanical properties of the product [11]. Some specific chemicals called biocides are used to prevent bacterial growth. The most common biocides are biguanides, quaternary ammonium salts, peroxides, alcohols, heavy metals, and halogens. These biocides use different mechanisms by which to inactivate bacteria. Quaternary ammonium compounds (QACs) are examples of cationic biocides [12,13], which target the inner membranes of microorganisms by ionic interactions with phospholipid components within the cytoplasm membrane, resulting in membrane disruption. The inactivation mechanism of the bacteria is electrostatic interaction between the positively charged N^+^ site and the negatively charged cell wall, which is a relatively slow mechanism [14]. QACs show the best antimicrobial activity when they have at least one long hydrocarbon chain of 8 to 19 carbon atoms substituted at the nitrogen end [15].

In the present study, a silane and quaternary ammonium functional copolymer series was synthesized to improve the properties of coated fabrics, such as washing fastness, light fastness, and biocidal activity. To reach this goal, silane functional monomers and quaternary functional methacrylate monomers were synthesized and used in emulsion polymerization. The surfaces of cotton fabrics were then coated with these copolymer solutions. The biocidal activities of the surfaces of coated fabrics were also tested against *Escherichia coli* and *Staphylococcus aureus*.

## 2. Materials and methods

### 2.1. Materials

3-(Acryloyloxy)-2-hydroxypropyl methacrylate (ACOM) (99%, Sigma Aldrich, St. Louis, MO, USA), 3-(aminopropyl)-triethoxysilane (APTES) (99%, Sigma Aldrich), 3,3′-iminobis-(N,N-dimethylpropylamine) (IDPA) (99%, Sigma Aldrich), 3-(dimethylamino)-1-propylamine (DMAPA) (99%, Sigma Aldrich), bromohexane (99%, Sigma Aldrich), and bromohexadecane (99%, Sigma Aldrich) were used as received without further purification. Dimethyl diallyl ammonium chloride (80%, Sigma Aldrich), ammonium persulfate (98%, Merck Millipore, Burlington, MA, USA), sodium dodecylbenzene sulfonate (technical grade, Sigma Aldrich), and nonionic emulsifier (Lutensol TDA-9, BASF Corporation, Ludwigshafen, Germany) were used as received without further purification for the polymerization process. Tryptic soy broth (TSB) and plate count agar (PCA) (both from Difco, BD Diagnostic Systems, Cockeysville, MD, USA) were used for the microbiological tests. *Staphylococcus aureus* (ATCC number: 25923) and *Escherichia coli* (ATCC number: 8739) were acquired from the Department of Molecular Biology and Genetics of Yıldız Technical University (İstanbul, Türkiye). All other materials used in this study were of reagent grade.

### 2.2. Methods

^1^H NMR (500 MHz) and ^13^C NMR (75 MHz) spectra were recorded using a Bruker Avance III 500 MHz spectrometer (Bruker, Billerica, MA, USA). The appropriate frequencies using either residual CDCl_3_ or DMSO-*d_6_* as an internal reference for ^1^H and ^13^C NMR were applied for the analysis of NMR data. A Spectrum One spectrometer (PerkinElmer, Waltham, MA, USA) was used for attenuated total reﬂectance Fourier transform infrared (ATR-FTIR) spectroscopy.

### 2.3. Synthesis of monomers

#### 2.3.1. Synthesis of methacrylate functional silane monomer (ACOM-APTES)

ACOM (12.8 g [0.065 mol]) was dissolved in 25 mL of chloroform in a round-bottom flask. The reaction medium was purified using nitrogen gas for 30 min. APTES (14.6 g [0.066 mol]) was mixed in 25 mL of chloroform in a separate vial.

After the ACOM solution was purified with nitrogen gas for 30 min, APTES solution was added dropwise for 2 h while stirring. Nitrogen gas was continuously used in the reaction vessel throughout the reaction period. The reaction continued at room temperature for 2 h. The product (ACOM-APTES) was obtained in a quantitative yield and was then characterized by NMR spectroscopy and FTIR spectroscopy after vacuum evaporation of the solvent.

#### 2.3.2. Synthesis of quaternary ammonium compounds

##### 2.3.2.1. Methacrylate functional bromohexadecane-based quaternary ammonium monomer (ACOM-Q1)

The ACOM-Q1 monomer (compound **1**) was synthesized according to the literature procedures [16]. ACOM (12 g [0.061 mol]) was added to the reaction vessel and dissolved by adding 20 mL of chloroform. The reaction medium was purified using nitrogen gas for 1–2 min. For the ACOM-Q1 monomer, IDPA (11.54 g [0.066 mol]) was added to a different vial and dissolved in 5 mL of chloroform. After the ACOM solution was purified with nitrogen gas for 1–2 min, the IDPA solution and ACOM-Q1 monomer were added dropwise to the ACOM solution for 1 h while stirring at room temperature. Nitrogen gas was injected into the reaction vessel throughout the reaction period, and the reaction continued at room temperature for 48 h. A viscous transparent solution was obtained in quantitative yield.

After the chloroform was evaporated, 10 g (0.025 mol) of compound **1**, 20.63 g (0.125 mol) of 5 equivalents of bromohexadecane, and 30 mL of acetonitrile were added to the reaction flask for quaternization. The reaction flask was placed in an oil bath at 60 °C for 48 h, after which it was removed and held on a rotary evaporator for 5 min to evaporate the acetonitrile. The acetonitrile was evaporated to about 5 mL and the product was precipitated over 200 mL of diethyl ether. A yellow orange oily solid (ACOM-Q1) was obtained at a yield of 60% by weight.

##### 2.3.2.2. Methacrylate functional bromohexane-based quaternary ammonium monomer (ACOM-Q2)

The ACOM-Q2 monomer (compound **2**) was synthesized according to the method used in the literature [16] using the same procedure as for ACOM-Q1. Here, quaternary ammonium functionality was obtained by using dimethylamino-1-propylamine (DMAPA) instead of IDPA. At the end of the reaction, a yellow-orange waxy solid (ACOM-Q2) was obtained at a yield of 70% by weight.

After the chloroform evaporated, 10 g (0.032 mol) of compound **2**, 26.08 g (0.165 mol) of 5 equivalents of bromohexane, and 30 mL of acetonitrile were added to the reaction flask. The reaction flask was placed in an oil bath at 60 °C for 48 h. After 48 h, the reaction flask was removed from the oil bath and held on a rotary evaporator for 5 min to evaporate a portion of the acetonitrile. The acetonitrile was evaporated to about 5 mL and the product was precipitated over 200 mL of diethyl ether. A yellow-orange solid (ACOM-Q2) was obtained at a yield of 68% by weight.

#### 2.3.3. Synthesis of methacrylate functional PDMS monomer (ACOM-PDMS)

ACOM (2.15 g [0.01 mol]) was dissolved in 20 mL of chloroform in a round-bottom flask. The reaction medium was purified with nitrogen gas for 30 min. PDMS (22 g [0.011 mol], molecular weight = 2000 g/mol) was mixed in 20 mL of chloroform in a separate vial.

After the ACOM solution was purified with nitrogen gas for 30 min, the PDMS solution was added dropwise to the ACOM solution for 2 h while stirring. The nitrogen gas was continuously used in the reaction vessel throughout the reaction period. The reaction continued at room temperature for 2 h.

ACOM-PDMS was obtained at a yield of 80% by weight and then characterized by NMR and FTIR techniques after evaporation under a vacuum.

### 2.4. Synthesis of emulsion copolymers

Table 1 shows the weight ratios of the monomers used in the emulsion copolymerization. Water and emulsifiers (10 mL, 8.35% by weight) were added to the reaction vessel and stirred under room temperature. The reflux condition was adjusted and nitrogen gas was purged for 15 min. The temperature was increased to within a range of 60 to 70 °C. As a typical example, synthesis of Recipe 1 was performed as follows: 8.35 g of emulsifiers (3.21 g of sodium dodecylbenzene sulfonate and 5.14 g of nonionic emulsifier [Lutensol TDA-9]) and 10 mL of water were added to the reaction vessel and stirred under room temperature. Subsequently, 3 g of ACOM-APTES, 3 g of ACOM-Q1, and 16 g of dimethyl diallyl ammonium chloride were added to the reaction vessel. Water was added to complete the formulation to a total of 100 g. The reaction temperature was increased to 60–70 °C. The free radical initiator APS (0.1% by weight) was dissolved in a small amount of water and added to the reaction medium in three portions at 30-min intervals. The reaction was then kept at the same temperature with reflux in a nitrogen atmosphere for 1 h while stirring. After 1 h, the reaction was completed by reducing the temperature to 30 °C. The total reaction time was approximately 150 min. After the polymerization was completed, the products were precipitated into isopropyl alcohol and filtered and dried for characterization using FTIR. Copolymers were obtained at a yield of 80%–85% by weight and then characterized by FTIR technique.

### 2.5. Cotton fabric coating

Initially, cotton fabric was dyed using a laboratory-type dyeing machine. For dyeing, reactive yellow 145 and reactive red 12 dyes were used on the mass of fabric at 1% and 3.5%, respectively.

In the dye bath, 80 g/L NaCl and 20 g/L Na_2_CO_3_ solutions were used. Dyeing continued for 1 h at 60 °C, after which the dyed fabric was neutralized, washed at room temperature, and rinsed thoroughly in tap water.

**Figure 1:**
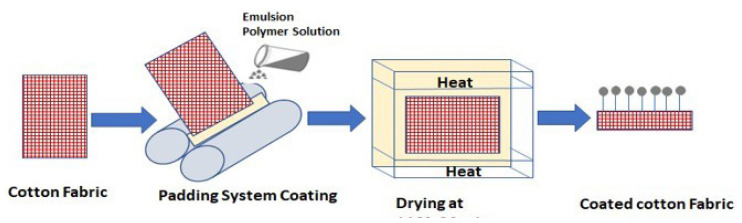
Coating of cotton fabric.

Solutions of emulsion polymers (Recipes 1 to 6) at three different concentrations were prepared for coating the dyed cotton fabric. Figure 1 shows the coating process. Typically, 20, 30, and 40 g/L solutions of emulsion copolymers were applied onto a fabric surface of 20 × 20 cm using the padding method and a laboratory double-roll padding with the squeezing rollers placed horizontally. The padding rate was 1–1.5 m/min with a squeezing roller pressure of 3.5 bar along the roller contact line. The padding was repeated twice. After each step, the fabric was dried in an oven at 110 °C for 30 min. Finally, various properties (BS EN ISO 105 X 12 Rubbing Fastness, ISO 105 C06 Washing Fastness, ISO 105 E01 Colour Fastness to Water, ISO 105 E01 Light Fastness) of the coated fabrics were investigated.

### 2.6. Biocidal activity

The biocidal activity of the fabrics was evaluated according to the ASTM E2149-13a standard test method. This test method was applied to measure the bactericidal activity of fabric samples containing antimicrobial agents under dynamic contact conditions. Briefly, 10 pieces of fabric of 1.0 ± 0.1 g treated with antimicrobial agents (Recipes 1 to 6) and similar untreated fabric samples were weighed and added to TSB flasks (50 ± 5 mL) containing 10^5^ CFU/mL bacteria. A broth medium flask containing only 10^5^ CFU/mL bacteria and no fabric sample was used as a control. At the beginning of the reaction (start time), 100 μL of sample was taken from each flask and spread on three PCA plates. All samples were then placed on a shaker incubator at 200 rpm and 37 °C, and 100 μL of samples were taken from those flasks at 4, 8, and 24 h and spread on PCA plates using serial dilutions. After the plates were incubated for 24 h at 37 °C, the colonies were counted, and the results were calculated using the following formulas:


$$\begin{array}{l} \text{log reduction}=\text{log}(\text{cell count of control})\text{-log}(\text{survivor count on test samples fabric materials})\\ \text{kill }\%=\frac{(\text{cells of control}-\text{survivor count on test samples fabric materials})\times 100}{\text{cell count of control}} \end{array}$$

## 3. Results and discussion

In the first part of this study, silane or quaternary amino functional methacrylate monomers were synthesized and characterized [17–22]. Emulsion copolymerization techniques were applied to obtain an acrylate latex formulation to coat cotton textile surfaces. Cation-bearing monomer units in the copolymer series also allowed for dyability as well as the antibacterial properties of the surface of the fabric, whereas the silane functionality enhanced the durability and washing cycle.

### 3.1. Methacrylate functional silane monomer (ACOM-APTES)

**Figure 2:**
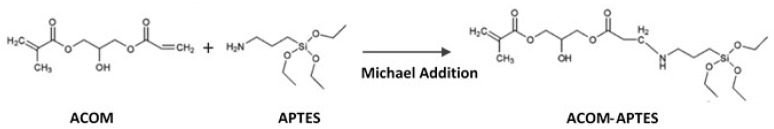
Synthesis of methacrylate functional monomer with silane groups on the side chain (ACOM-APTES).

**Figure 3:**
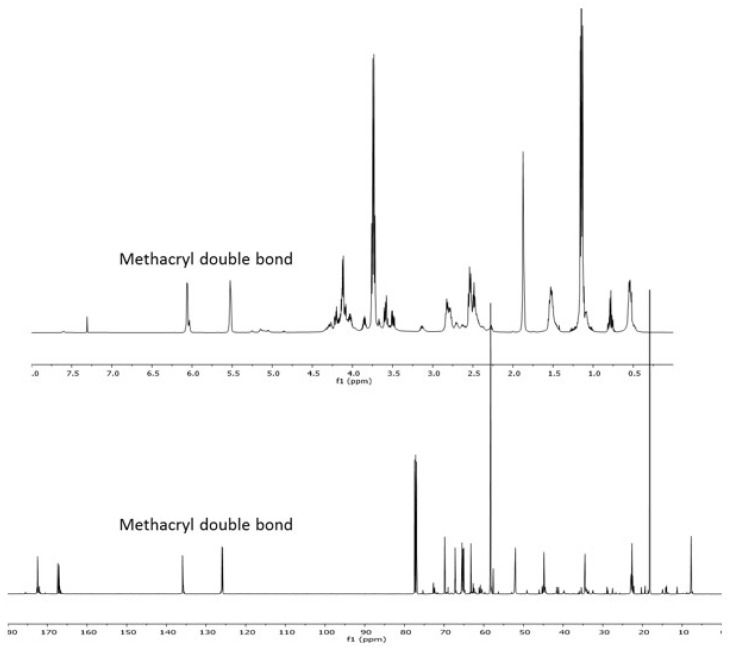
^1^H NMR (top) and ^13^C NMR (bottom) spectra of ACOM-APTES.

Methacrylate functional monomers with silane groups on the side chain were synthesized as shown in Figure 2. Because the amine can act as both the donor agent and base, the addition of a catalyst is often not necessary. APTES reacts with an acrylate double bond through the Michael addition pathway at room temperature without the addition of a catalyst [23]. The product was a clear viscous liquid. The product was characterized by NMR and FTIR spectroscopy. The ^1^H NMR spectrum showed the characteristic methacrylate protons (H_2_C = C-[CH_3_]-) at 5.1 and 6.0 ppm, and new silanol peaks (-Si-OCH_2_CH_3_) were observed at 1 ppm (Figure 3). Complete disappearance of acrylate double bonds at 5.70, 6.16, and 6.32 ppm was observed.

The ACOM-APTES product was also characterized by ^13^C NMR analysis as shown in Figure 3. Ester carbonyl was observed at 170 ppm, and new carbon peaks (-NH-CH_2_-) were observed at 51.9 and 51.5 ppm. Methacrylate double bonds (-C=C[CH_3_]-) were observed at 135 ppm. It should be noted that the ACOM consisted of two isomers formed by 1,2-glycerol diester and 1,3-glycerol diester with different ratios of 20% minor isomer; therefore, an isomeric mixture of ACOM and APTES was obtained.

The ACOM-APTES product was also characterized using FTIR. The FTIR spectrum showed the characteristic methacrylate double bonds at 1630 cm^−1^. The peak at 1720 cm^−1^ showed carbonyl stretching. Characteristic peaks for silane groups were observed at 1079 cm^−1^ (Si-O-CH_3_ stretching), 1260–1259 cm^−1^ (CH_3_ degradation in Si-CH_3_), and 2950–2960 cm^−1^ (asymmetric CH_3_ stretching in Si-CH_3_).

### 3.2. Methacrylate functional IDPA-based quaternary monomers (ACOM-Q1)

**Figure 4:**
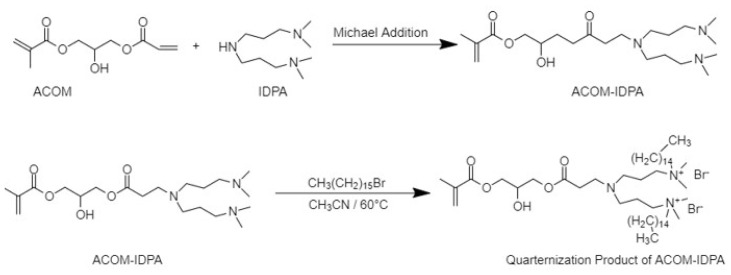
Synthesis and quaternization of methacrylate functional (IDPA) monomer (ACOM-Q1).

ACOM-Q1 was synthesized according to the method in the literature. A new type of amine monomer was synthesized using the Michael addition reaction between IDPA and ACOM at room temperature as shown in Figure 4. The secondary amine product was obtained using equimolar amounts of reactants and without the use of a catalyst.

**Figure 5:**
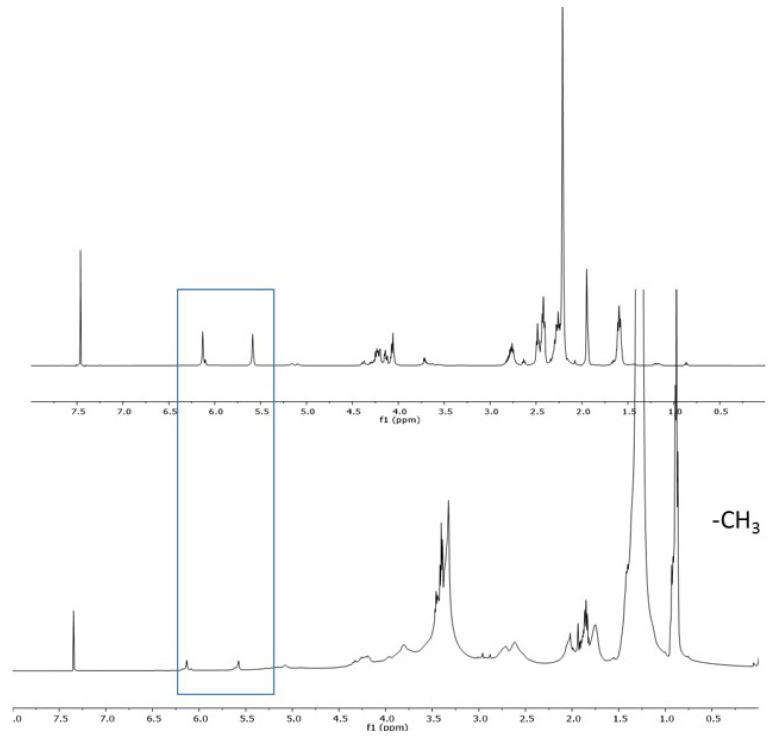
^1^H NMR spectrum of ACOM-IDPA (top) and quaternized ACOM-IDPA (bottom).

The monomer structures were confirmed using FTIR and NMR (^1^H and ^13^C). Figure 5 shows the ^1^H and ^13^C NMR spectra of ACOM-IDPA and quaternized ACOM-IDPA.

The ^1^H NMR spectrum showed methacrylate protons at 5.5 and 6.0 ppm. Methyl protons of IDPA (-N-CH_3_) were observed at 2.2 ppm as singlets. The ACOM-IDPA product was similarly characterized by ^13^C NMR analysis. The ^13^C NMR spectrum showing the methyl carbons attached to nitrogen (-N-CH_3_) appeared at 45.3 ppm. The FTIR spectrum of this product showed the characteristic absorptions of -OH and -NH, C=O, and C=C groups at 3500, 1722, and 1637 cm^−1^, respectively.

After characterization of ACOM-IDPA, the product was quaternized with excess bromohexadecane in dry acetonitrile at 60 °C (Figure 4). The quaternized product was characterized using ^1^H NMR, ^13^C NMR, and FTIR techniques. Typically, introduced alkyl units were observed at 0.8–1.2 ppm for the -CH_2_ and -CH_3_ protons. Quaternized methyl protons (-N^+^[CH_3_]_2_) appeared at 3.5 ppm. It should also be noted that methacrylate protons of ACOM-IDPA were present after quaternization (Figure 5).

The ^13^C NMR spectrum of the bromohexadecane derivative showed the characteristic peak for ^+^N-CH_2_-CH_2_ at 64.5 ppm. Terminal methyl carbons of hexadecyl bromide units were observed at 15.5 ppm. FTIR showed quaternary N-C stretching at 1261 cm^−1^ and methacrylate double bond stretching at 1637 cm^−1^.

### 3.3. Methacrylate functional DMAPA-based quaternary monomers (ACOM-Q2)

**Figure 6:**
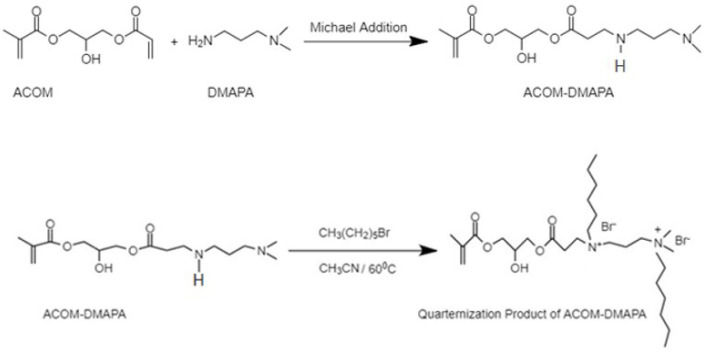
Synthesis and quaternization of methacrylate functional (DMAPA) monomer (ACOM-Q2).

The Michael addition reaction between DMAPA and ACOM was conducted at room temperature (Figure 6).

**Figure 7:**
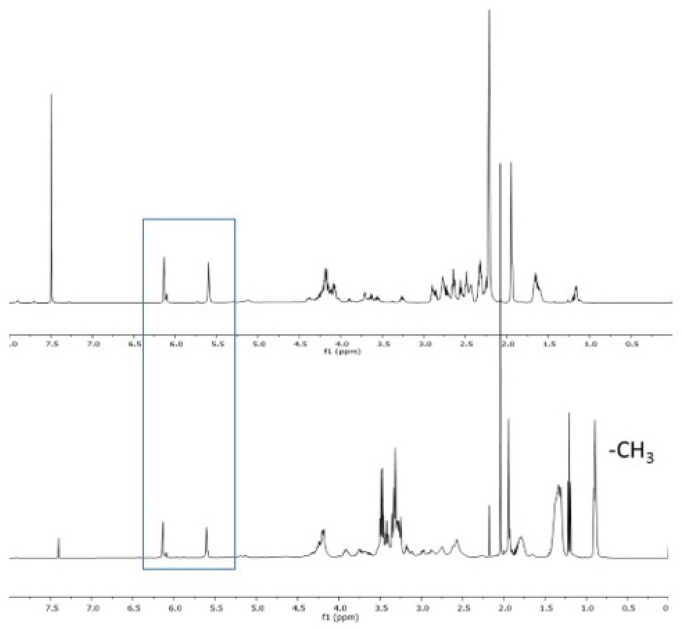
^1^H NMR spectrum of ACOM-DMAPA (top) and quaternized ACOM-DMAPA (bottom).

FTIR and NMR (^1^H and ^13^C) techniques were used to confirm the structures of the monomer. ACOM-DMAPA indicated methacrylate protons at 5.5 and 6.1 ppm and the disappearance of acrylate vinyl peaks confirmed the formation of the Michael adduct. After quaternization with bromohexane, these protons were still present and were not consumed during the reaction. Newly formed methyl protons of hexyl units (−CH_3_) were observed at 0.85 ppm, as shown in Figure 7. The ^13^C NMR spectrum was also indicative of the quaternized product. After quaternization, the carbon attached to nitrogen (NH-CH_2_-) shifted from 45 to 51 ppm.

### 3.4. Methacrylate functional PDMS monomer (ACOM-PDMS)

**Figure 8:**
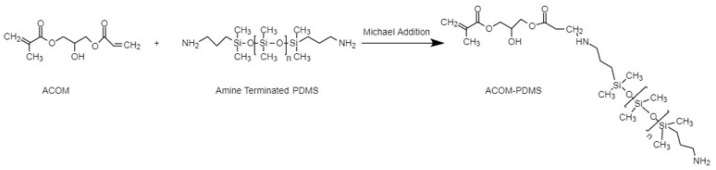
Synthesis of methacrylate functional monomer with amine-terminated PDMS.

Methacrylate functional monomers with PDMS groups were synthesized as shown in Figure 8. Amine-terminated PDMS reacted with an acrylate double bond through the Michael addition pathway at room temperature, and the reaction was complete in 2 h. An excess amount of amine-terminated PDMS was used to prevent the addition of ACOM to PDMS.

**Figure 9:**
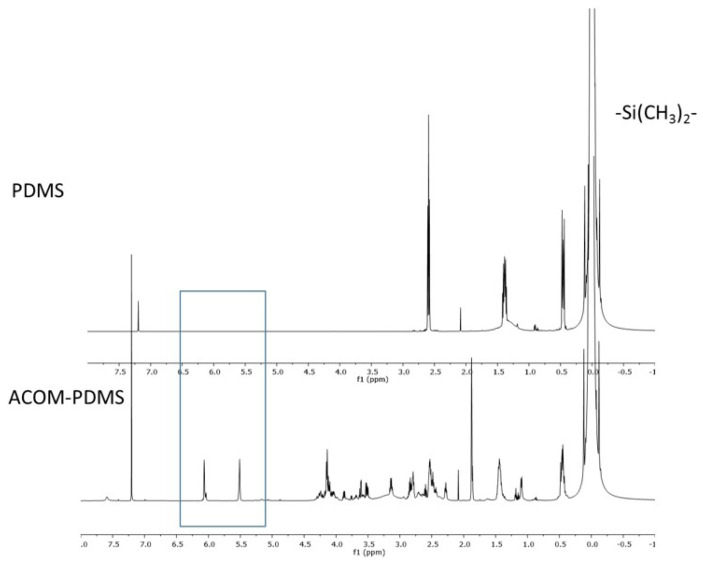
^1^H NMR spectrum of PDMS (top) and ACOM-PDMS (bottom).

The product was characterized by NMR and FTIR. In the ^1^H NMR spectrum (Figure 9), methyl protons of the PDMS unit were observed at δ 0.05 ppm. The methylene protons of NH_2_-CH_2_- and -CH_2_-Si(CH_3_)_2_- appeared at 2.5 ppm and 0.51 ppm, respectively. After the Michael addition to the acrylate part, the ACOM acrylate proton peaks disappeared, while the methacrylate double bond protons observed at 5.5 and 6.0 ppm held their intensity. FTIR analysis was also used in the characterization of the product. The peak at 1265 cm^−1^ belonged to the bending vibration absorption of -CH_3_ in PDMS, the split peaks at 1100 and 1020 cm^−1^ were respectively ascribed to the absorptions of dissymmetric and symmetric stretching vibrations of the group of Si-O-Si, and the peak at 800 cm^−1^ was ascribed to the helical conformation vibration absorption of Si-O groups in PDMS. The peak at 1689 cm^−1^ belonged to the bending vibration absorption of C=C in ACOM.

### 3.5. Emulsion polymerization

**Figure 10:**
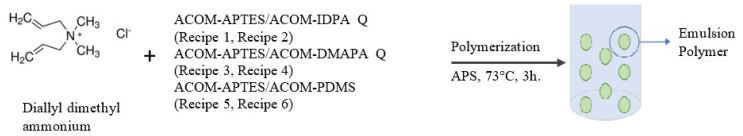
Typical formulations for emulsion polymerization.

**Figure 11:**
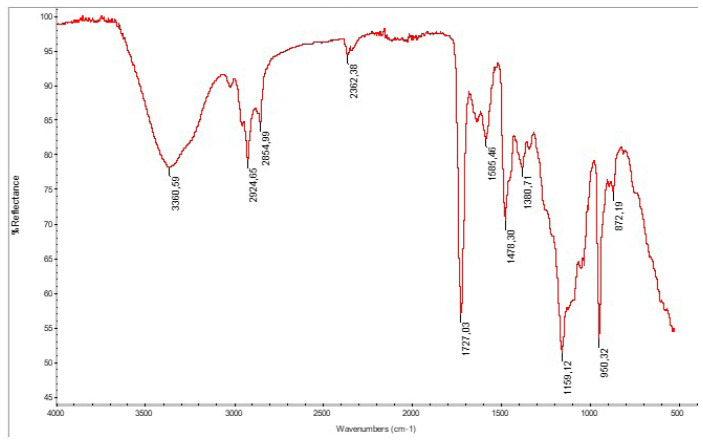
Fourier transform infrared (FTIR) spectrum of emulsion polymer for Recipe 1.

The monomers used in the continuous emulsion polymerization are given in Table 1 and a representative schematic formulation is given in Figure 10. Dimethyl diallyl ammonium chloride is a cationic monomer that can form a cationic water-soluble polymer through free radical polymerization and continuous emulsion polymerization techniques. Dimethyl diallyl ammonium chloride and methacrylate functional monomers were polymerized using emulsifying agents at 60–70 °C. Ammonium persulfate was used as an initiator and was introduced into the reaction medium in three parts. After the polymerization was completed, the products were precipitated into isopropyl alcohol and filtered and dried for characterization using FTIR. In a typical example, the FTIR spectrum of the emulsion polymer for Recipe 1 showed the characteristic absorption peaks of O-H and N-H, C-H stretching, and C=O and C=C groups at 3360, 2854–2924 doublet peaks, 1727, and 1658 cm^−1^, respectively, and Si-O-Si groups at 1159 and 950 cm^−1^ (Figure 11).

The zeta potential value was assessed, and particle size measurements were conducted directly from the recipe solutions using the DLS technique. Table 2 shows the particle sizes and zeta potential values of the emulsion polymerizations. Particle size values of emulsion polymerizations with ACOM-PDMS monomers (Recipes 5 and 6) were 115–128 nm, being larger than the others. The presence of PDMS with a high molecular weight resulted in an increase in the particle size of the droplets. The emulsion polymerizations with ACOM-Q1 (Recipes 1 and 2) and ACOM-Q2 (Recipes 3 and 4) monomers had smaller particles. The emulsion polymerizations with ACOM-Q1 and ACOM-Q2 monomers had smaller particles and more cationic characters than the emulsion polymers with ACOM-PDMS because they had quaternary amine groups. The presence of PDMS with a high molecular weight resulted in an increase in the particle size of the droplets.

Using ACOM-Q2 in the emulsion formulation, Recipes 3 and 4 gave higher ζ-potential of 41.0 and 40.8 mV, respectively. Increasing the hydrophobicity of the monomer unit by using hexadecyl bromide (Recipes 1 and 2) caused a decrease in the ζ-potential to 36.8 and 35.2 mV, respectively. Incorporation of PDMS also decreased the ζ-potential of emulsion to <20 mV. It seems that the hydrodynamic shear surrounding the colloidal particles prevents the development of electrical charge at the interface between the colloid surface and the water medium. Lower zeta potential values with the hydrophobic nature of the colloids indicates drops in the long-term stability of the colloid dispersion and a tendency of aggregation [24]. Thus, it is expected that the long-term stability of Recipe 1 and Recipe 2 is better than that of the other formulations (Recipe 3 to Recipe 6).

### 3.6. Cotton fabric coating

Table 3 summarizes the fastness properties of the fabrics after coating. It provides the rubbing, washing, water, and light fastness values of the fabrics coated with copolymers. Comparing the bromohexane unit-bearing polymer (Recipe 1) and the polymer with the bromohexadecane unit (Recipe 3), it was observed that Recipe 3 produced relatively better rubbing and washing fastness properties than the other recipes. The rubbing and washing fastness values of the polymers made with ACOM-PDMS monomer (Recipes 5 and 6) were better than those of the other polymers. An increase was observed in the fastness values obtained with the use of chemicals at increasing concentrations. None of the polymers lowered the water and light fastness values of the fabrics to which they were applied. It was observed that with an increase in silane groups, the covalent bonding to the textile surface increased the washing fastness.

### 3.7. Biocidal activity

The killing efficacy of the fabric samples was tested against two significant pathogens: the gram-negative bacterium *E. coli* (ATCC number: 25922) and the gram-positive bacterium *S. aureus* (ATCC number: 25923). Test samples were treated with these two microorganisms at a concentration of approximately 1.5–3.0 × 10^5^ CFU cm^−2^, and reductions in cell number were noted at specific time intervals (0, 2, 4, 8, and 24 h).

**Figure 12:**
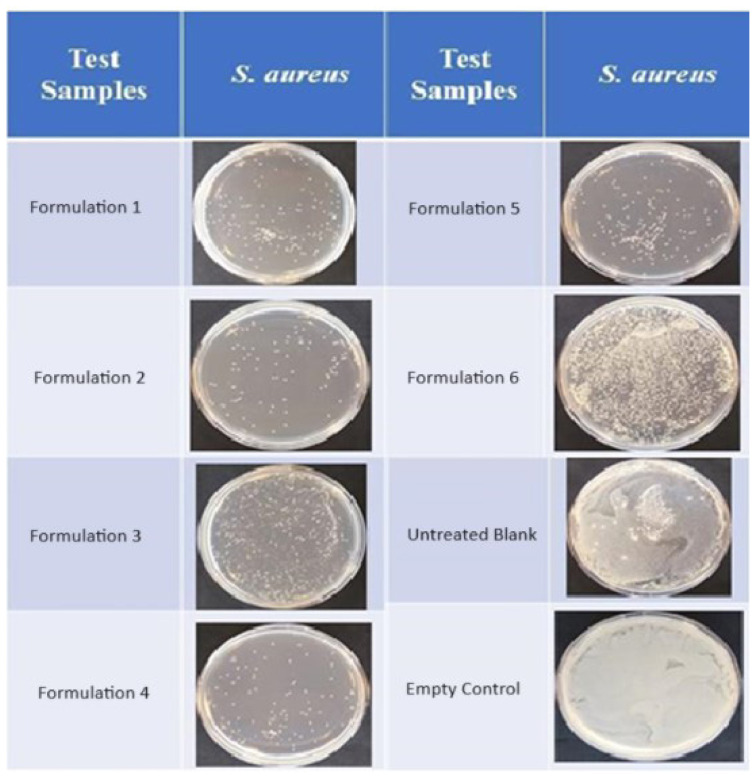
Petri images of surviving bacteria after exposure of *Staphylococcus aureus* to test samples for 24 h (these samples were diluted by 10^−2^).

**Figure 13:**
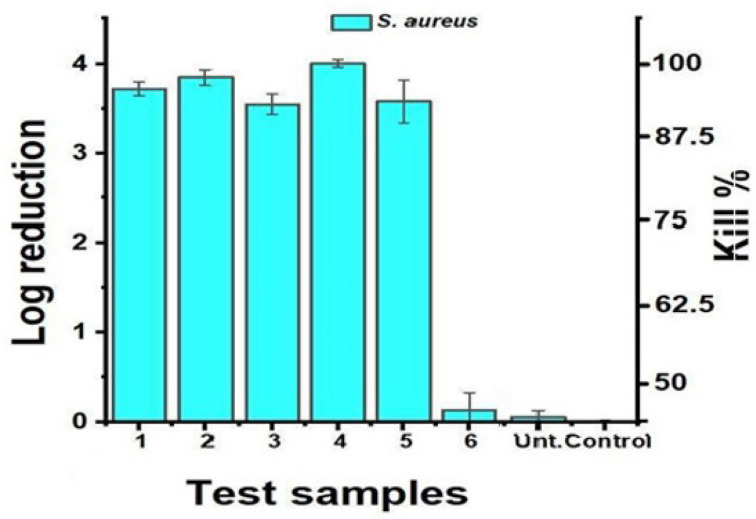
Results of log reduction and kill % for *Staphylococcus aureus* by test samples at 24 h.

As can be seen in Figures 12 and 13, the test samples showed antibacterial activity against *S. aureus* but were ineffective against *E. coli*. It was observed that the bactericidal activity against *S. aureus* of the samples produced from Recipes 1 to 6, as shown in Figure 12, was quite high and lasted for 24 h; the values of log reduction were 3.72, 3.85, 3.55, 4.01, and 3.58 at 24 h, respectively, for the first five formulations. For Recipe 6, it was observed that the inhibition effect decreased after 8 h (the values of log reduction were 0.14, 0.34, and 0.29, at 24 h, respectively). In petri images, the difference between the test and control samples (only bacteria and/or untreated samples) was quite evident; therefore, it could be promising to test Recipes 1 through 6 to prevent infections caused by *S. aureus* in the presence of open wounds, such as injuries and burns or due to chronic diseases, such as diabetes, hidradenitis, or dermatitis [25]. However, it was observed that the test samples did not have bactericidal activity against *E. coli*, and their bacteriostatic effects disappeared within the first 4 h (data not shown). Presumably, the difference in cell walls between the two organisms and the resistance potential of *E. coli* caused this difference in activity.

## 4. Conclusion

In the present study, silane- and quaternary ammonium-bearing copolymers were synthesized using continuous emulsion polymerization techniques. First, different types of methacrylate monomers having hydrophobic and quaternary groups were synthesized and characterized. The particle size of the droplets was within the range of 78–125 nm. PDMS increased the particle size of the droplets and decreased the zeta potential values. Recipes prepared at different concentrations were then used for coating cotton fabric. Although untreated fabric had poor fastness values, the fastness properties of the coated fabrics were improved. The PDMS-bearing formulation (i.e., Recipe 6) produced high fastness values. It was observed that all the polymer-coated cotton fabrics showed biocidal activity of >90% against *S. aureus*; however, the presence of quaternary ammonium hexadecyl units on the textile surface produced the highest killing efficiency against that bacterium (i.e., >99%). It appears that coating textile products such as socks, sheets, and bandages with these polymers could help prevent nosocomial infections caused by *S. aureus*.

## Figures and Tables

**Table 1:** Monomer weight ( % ) used in emulsion copolymerization.^a^

	Recipe 1	Recipe 2	Recipe 3	Recipe 4	Recipe 5	Recipe 6
ACOM -APTES	3	2	3	2	3	2
ACOM - Q1	3	4	-	-	-	-
ACOM - Q2	-	-	3	4	-	-
ACOM -PDMS	-	-	-	-	3	4

a The other constant additives were emulsifiers (8.35% by weight), dimethyl diallyl ammonium chloride (16% by weight), free radical initiator APS (0.1% by weight), and water in the formulations.

**Table 2:** Solid content and DLS analysis of emulsion polymers.

	Solid Content ( % )	Zeta Potential ( mV )	Particle Size ( d.nm )
Recipe 1	28.3	36.8	78
Recipe 2	28.7	35.2	81
Recipe 3	29.1	41.0	89
Recipe 4	28.8	40.8	85
Recipe 5	28.4	12.5	115
Recipe 6	28.2	17.2	128

**Table 3:** Fastness properties of fabrics after coating.

	Recipe 1			Recipe 2		
	20 g/L	30 g/L	40 g/L	20 g/L	30 g/L	40 g/L
Rubbing fastness	3	3	3	2	2	3
Washing fastness	2	3	3	1	2	2
Water color Fastness	4	4	4	4	4	4
Light fastness	3	3	3	3	3	3
	**Recipe 3**			**Recipe 4**		
	**20 g/L**	**30 g/L**	**40 g/L**	**20 g/L**	**30 g/L**	**40 g/L**
Rubbing fastness	3	3	4	2	2	3
Washing fastness	3	3	4	2	2	3
Water color Fastness	4	4	4	4	4	4
Light fastness	3	3	3	3	3	3
	**Recipe 5**			**Recipe 6**		
	**20 g/L**	**30 g/L**	**40 g/L**	**20 g/L**	**30 g/L**	**40 g/L**
Rubbing fastness	3	3	4	4	4	5
Washing fastness	3	4	5	4	5	5
Water color Fastness	4	4	4	4	4	4
Light fastness	3	3	3	3	3	3

5 = Very good, 4 = good, 3= moderate, 2 = worse, 1 = bad (untreated dyed fabric: 1 point for rubbing fastness value, 2 points for washing fastness value, 4 points for water fastness value, 3 points for light fastness value).
